# 19β,28-Ep­oxy-18α-olean-3β-ol

**DOI:** 10.1107/S1600536809030311

**Published:** 2009-08-08

**Authors:** R. C. Santos, R. M. A. Pinto, A. Matos Beja, J. A. R. Salvador, J. A. Paixão

**Affiliations:** a Laboratório de Química Farmacêutica, Faculdade de Farmácia, Universidade de Coimbra, P-3000-548 Coimbra, Portugal; b CEMDRX, Departamento de Física, Faculdade de Ciências e Tecnologia, Universidade de Coimbra, P-3004-516 Coimbra, Portugal

## Abstract

The title triterpene, C_30_H_50_O_2_, is an 18α-oleanane derivative prepared by the Wagner–Meerwein rearrangement of betulin with Bi(OTf)_3_.*x*H_2_O (OTF is trifluoromethanesulfonate). There are two symmetry-independent mol­ecules in the asymmetric unit that show no significant differences concerning bond lengths and angles. The conformation of the six-membered rings is close to a chair form, while the five-membered epoxide rings adopt envelope conformations. All rings are *trans*-fused. In the crystal, mol­ecules are held together by O—H⋯O hydrogen bonds. A quantum-mechanical *ab initio* Roothan Hartree–Fock calculation on the isolated mol­ecule gives values for bond lengths and valency angles close to the experimental values. The calculations also reproduce well the mol­ecular conformation with calculated puckering parameters that match well the observed values.

## Related literature

For terpene rearrangements, see: King *et al.* (1968 ▶). For Wagner–Meerwein rearrangements, see: Hanson (1991 ▶). For the synthesis of 18α-oleanane derivatives, see: Salvador *et al.* (2009 ▶). For the cytotoxic activity of 18α-oleanane derivatives, see: Urban *et al.* (2007 ▶); Thibeault *et al.* (2007 ▶). For puckering parameters, see: Cremer & Pople (1975 ▶) and for asymmetry parameters, see: Duax & Norton (1975 ▶). For the program *GAMMESS* used to perform the quantum chemical calculations, see: Schmidt *et al.* (1993 ▶).
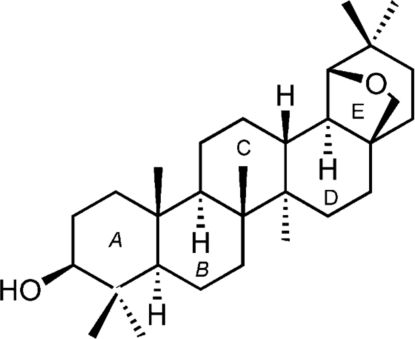


## Experimental

### 

#### Crystal data

C_30_H_50_O_2_
*M* *_r_* = 442.70
Monoclinic, 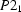

*a* = 13.2824 (2) Å
*b* = 12.6702 (2) Å
*c* = 15.5236 (3) Å
β = 94.9990 (10)°
*V* = 2602.54 (8) Å^3^
*Z* = 4
Mo *K*α radiation
μ = 0.07 mm^−1^
*T* = 293 K
0.40 × 0.25 × 0.18 mm

#### Data collection

Bruker APEXII CCD area-detector diffractometer
Absorption correction: multi-scan (*SADABS*; Sheldrick, 2000 ▶) *T*_min_ = 0.930, *T*_max_ = 0.998
63144 measured reflections
6510 independent reflections
5427 reflections with *I* > 2σ(*I*)
*R*_int_ = 0.039

#### Refinement

*R*[*F* ^2^ > 2σ(*F* ^2^)] = 0.048
*wR*(*F* ^2^) = 0.140
*S* = 1.02
6510 reflections
593 parameters
1 restraint
H-atom parameters constrained
Δρ_max_ = 0.28 e Å^−3^
Δρ_min_ = −0.19 e Å^−3^

### 

Data collection: *SMART* (Bruker, 2003 ▶); cell refinement: *SAINT* (Bruker, 2003 ▶); data reduction: *SAINT*; program(s) used to solve structure: *SHELXS97* (Sheldrick, 2008 ▶); program(s) used to refine structure: *SHELXL97* (Sheldrick, 2008 ▶); molecular graphics: *PLATON* (Spek, 2009 ▶); software used to prepare material for publication: *SHELXL97*.

## Supplementary Material

Crystal structure: contains datablocks global, I. DOI: 10.1107/S1600536809030311/bt5024sup1.cif

Structure factors: contains datablocks I. DOI: 10.1107/S1600536809030311/bt5024Isup2.hkl

Additional supplementary materials:  crystallographic information; 3D view; checkCIF report

## Figures and Tables

**Table 1:** Hydrogen-bond geometry (Å, °)

*D*—H⋯*A*	*D*—H	H⋯*A*	*D*⋯*A*	*D*—H⋯*A*
O3*A*—H3*A*1⋯O19*A*^i^	0.82	2.04	2.853 (3)	171
O3*B*—H3*B*1⋯O3*A*^ii^	0.82	2.12	2.920 (3)	164

Symmetry codes: (i) 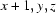; (ii) 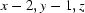.

**Fig. 1.**
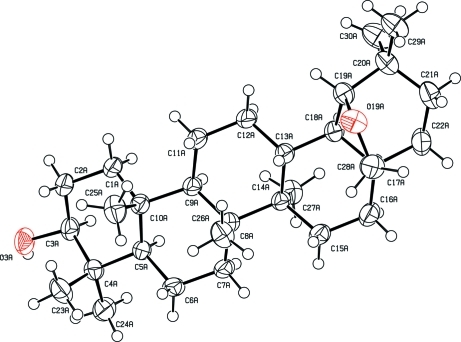
ORTEPII plot of one of the two molecules in the asymmetric unit. Displacement ellipsoids are drawn at the 50% level.

## supplementary crystallographic information

### Comment

Terpenes were among those natural products wherein rearrangements were earliest detected and studied (King *et al.*, 1968). Rearrangements involving the formation of a carbocation and 1,2-migrations of hydrogen or alkyl groups from one carbon to a neighboring carbon are designated as Wagner–Meerwein rearrangements (Hanson, 1991). This group of transformations constitutes a particularly important field of research in organic chemistry and has been widely reported in triterpenoid chemistry. Recently, we have reported the use of bismuth(III) salts as catalysts for the Wagner-Meerwein rearrangement of lupane derivatives with expansion of ring E and formation of an additional O-containing ring (Salvador *et al.*, 2009). Using this procedure, the Wagner-Meerwein rearrangement of betulin afforded the title compound in high yield.

In this communication we report the molecular structure of the 19β,28-Epoxy-18α-olean-3β-ol (I), determined by single-crystal X-ray diffraction, and compare it with that of the free molecule as given by quantum mechanical *ab initio* calculation.

The structure of compound (I) with the corresponding atomic numbering scheme is shown in Fig. 1. A l l bond lengths and valency angles have typical values for this type of compounds. All rings are fused *trans* as shown by the angle between the least-squares planes of the rings [molecule A: rings A and B: 16.27 (12)°, B and C: 9.60 (12)°, C and D: 0.40 (12)°, D and E: 15.59 (13)°; molecule B: rings A and B: 13.68 (15)°, B and C: 10.74 (14)°, C and D: 2.78 (13)°, D and E: 13.54 (16)°]. Rings A to E have conformations close to chair as shown by the Cremer & Pople (1975) parameters [Molecule A: ring A: Q = 0.571 (3) Å, θ = 2.1 (3)° and φ = 79 (6)°; B: Q = 0.575 (3) Å, θ = 9.2 (3)° and φ = 17.5 (16)°; C: Q = 0.599 (3) Å, θ = 6.2 (3)° and φ = 336 (2)°; D: Q = 0.521 (3) Å, θ = 168.4 (3)° and φ = 243.2 (14)°; E: Q = 0.661 (3) Å, θ = 21.6 (3)° and φ = 59.1 (7)°; Molecule B: ring A: Q = 0.553 (4) Å, θ = 4.6 (4)° and φ = 61 (5)°; B: Q = 0.569 (3) Å, θ = 7.5 (3)° and φ = 11 (2)°; C: Q = 0.585 (3) Å, θ = 4.2 (3)° and φ = 30 (3)°; D: Q = 0.537 (3) Å, θ = 170.0 (3)° and φ = 236.5 (17)°; E: Q = 0.656 (3) Å, θ = 18.9 (3)° and φ = 61.6 (10)°; The epoxyde-ring has a C18-envelope conformation [molecule A: q_2_ = 0.461 (3)Å and φ_2_ = 255.3 (3)° and asymmetry parameters (Duax & Norton, 1975) ΔC_s_(C18A) = ΔC_s_(C28A,O19A) = 2.1 (2)° molecule B: q_2_ = 0.468 (3)Å and φ_2_ = 254.6 (4)° and asymmetry parameters (Duax & Norton, 1975) ΔC_s_(C18B) = ΔC_s_(C28B,O19B) = 1.6 (3)°].

The molecules are hydrogen bonded involving the hydroxyl and epoxyde groups. The hydroxyl group of molecule A acts as a donor towards a neighbour hydroxyl group of a neighbour A molecule acting as an acceptor forming a chain of hydrogen bonds running along the *a* axis. The hydroxyl group of molecule B acts as a donor towards the epoxyde group of a neighbour A molecule.

In order to gain some insight on how the crystal packing of (I) might affect the molecular geometry we have performed a quantum chemical calculation on the equilibrium geometry of the free molecule. These calculations were performed with the computer program GAMMESS (Schmidt *et al.*, 1993). A molecular orbital Roothan Hartree-Fock method was used with an extended 6–31 G(d,p) basis set. Tight conditions for convergence of both the self-consistent field cycles and maximum density and energy gradient variations were imposed (10^-6^atomic units). The program was run on the Milipeia cluster of UC-LCA (using 16 Opteron cores, 2.2 GHz runing Linux).

The *ab*-*initio* calculations reproduce well the observed experimental bond length and valency angles of the molecule. Also, the calculated conformation of the rings are very close to the experimental values, with the exception of ring C for which the calculations gave a conformation closer to the ideal chair than experiment.

### Experimental

The synthesis of the 19β,28-epoxy-18α-olean-3β-ol was efficiently acomplished by Wagner-Meerwein rearrangement of commercially available betulin with Bi(OTf)_3_.xH_2_O in CH_2_Cl_2_ at reflux (Salvador *et al.*, 2009). The product of this reaction was isolated in 95% yield and identified as the title compound from IR, ^1^H and ^13^C NMR spectroscopy data (Salvador *et al.*,2009). Recrystallization from ethanol at room temperature gave colourless single crystals suitable for X-ray diffraction analysis.

### Refinement

All hydrogen atoms were refined as riding on their parent atoms using *SHELXL97* defaults except for that of the hydroxyl group which had its coordinates freely refined with *U*_iso_= 1.5 *U*_eq_ of the O atoms.

The absolute configuration was known from the synthetic route, but could not be determined from the X-ray data. Thus,  Friedel pairs were merged for refinement.

### Crystal data

**Table:**

C_30_H_50_O_2_	*F*(000) = 984
*M**_r_* = 442.70	*D*_x_ = 1.130 Mg m^−^^3^
Monoclinic, *P*2_1_	Mo *K*α radiation, λ = 0.71073 Å
Hall symbol: P 2yb	Cell parameters from 9126 reflections
*a* = 13.2824 (2) Å	θ = 2.5–27.5°
*b* = 12.6702 (2) Å	µ = 0.07 mm^−^^1^
*c* = 15.5236 (3) Å	*T* = 293 K
β = 94.999 (1)°	Block, clear colourless
*V* = 2602.54 (8) Å^3^	0.40 × 0.25 × 0.18 mm
*Z* = 4	

### Data collection

**Table:**

Bruker APEXII CCD area-detector diffractometer	6510 independent reflections
Radiation source: fine-focus sealed tube	5427 reflections with *I* > 2σ(*I*)
graphite	*R*_int_ = 0.039
φ and ω scans	θ_max_ = 27.9°, θ_min_ = 1.5°
Absorption correction: multi-scan (*SADABS*; Sheldrick, 2000)	*h* = −17→17
*T*_min_ = 0.930, *T*_max_ = 0.998	*k* = −16→16
63144 measured reflections	*l* = −20→20

### Refinement

**Table:**

Refinement on *F*^2^	Primary atom site location: structure-invariant direct methods
Least-squares matrix: full	Secondary atom site location: difference Fourier map
*R*[*F*^2^ > 2σ(*F*^2^)] = 0.048	Hydrogen site location: inferred from neighbouring sites
*wR*(*F*^2^) = 0.140	H-atom parameters constrained
*S* = 1.02	*w* = 1/[σ^2^(*F*_o_^2^) + (0.0834*P*)^2^ + 0.4115*P*] where *P* = (*F*_o_^2^ + 2*F*_c_^2^)/3
6510 reflections	(Δ/σ)_max_ = 0.001
593 parameters	Δρ_max_ = 0.28 e Å^−^^3^
1 restraint	Δρ_min_ = −0.19 e Å^−^^3^

### Special details

**Table:**

Geometry. All e.s.d.'s (except the e.s.d. in the dihedral angle between two l.s. planes) are estimated using the full covariance matrix. The cell e.s.d.'s are taken into account individually in the estimation of e.s.d.'s in distances, angles and torsion angles; correlations between e.s.d.'s in cell parameters are only used when they are defined by crystal symmetry. An approximate (isotropic) treatment of cell e.s.d.'s is used for estimating e.s.d.'s involving l.s. planes.
Refinement. Refinement of *F*^2^ against ALL reflections. The weighted *R*-factor *wR* and goodness of fit *S* are based on *F*^2^, conventional *R*-factors *R* are based on *F*, with *F* set to zero for negative *F*^2^. The threshold expression of *F*^2^ > σ(*F*^2^) is used only for calculating *R*-factors(gt) *etc*. and is not relevant to the choice of reflections for refinement. *R*-factors based on *F*^2^ are statistically about twice as large as those based on *F*, and *R*- factors based on ALL data will be even larger.

### Fractional atomic coordinates and isotropic or equivalent isotropic displacement parameters (Å^2^)

**Table:**

	*x*	*y*	*z*	*U*_iso_*/*U*_eq_	
C1A	1.21538 (19)	1.06069 (19)	0.31109 (18)	0.0404 (5)	
H1A1	1.1745	1.1233	0.2993	0.049*	
H1A2	1.2266	1.0531	0.3734	0.049*	
C2A	1.3177 (2)	1.0762 (2)	0.27390 (19)	0.0436 (6)	
H2A1	1.3071	1.0887	0.2121	0.052*	
H2A2	1.3515	1.1374	0.3004	0.052*	
C3A	1.38282 (17)	0.9802 (2)	0.29068 (16)	0.0380 (5)	
H3A	1.3919	0.9699	0.3534	0.046*	
C4A	1.33528 (17)	0.8782 (2)	0.25066 (16)	0.0362 (5)	
C23A	1.3342 (2)	0.8791 (3)	0.15174 (18)	0.0536 (7)	
H23A	1.4024	0.8802	0.1357	0.080*	
H23B	1.2991	0.9407	0.1291	0.080*	
H23C	1.3006	0.8170	0.1285	0.080*	
C24A	1.40181 (19)	0.7852 (2)	0.2846 (2)	0.0509 (7)	
H24A	1.3767	0.7210	0.2578	0.076*	
H24B	1.4002	0.7798	0.3462	0.076*	
H24C	1.4701	0.7967	0.2710	0.076*	
C5A	1.22947 (16)	0.86733 (18)	0.28626 (14)	0.0309 (4)	
H5A	1.2447	0.8626	0.3491	0.037*	
C6A	1.17428 (18)	0.7645 (2)	0.26226 (18)	0.0408 (6)	
H6A1	1.1449	0.7681	0.2029	0.049*	
H6A2	1.2218	0.7062	0.2669	0.049*	
C7A	1.09128 (18)	0.74588 (19)	0.32233 (19)	0.0410 (5)	
H7A1	1.1221	0.7385	0.3810	0.049*	
H7A2	1.0575	0.6799	0.3065	0.049*	
C8A	1.01189 (16)	0.83426 (18)	0.32031 (15)	0.0331 (5)	
C26A	0.94773 (19)	0.8264 (3)	0.23252 (17)	0.0479 (6)	
H26A	0.9915	0.8266	0.1865	0.072*	
H26B	0.9025	0.8855	0.2260	0.072*	
H26C	0.9093	0.7621	0.2306	0.072*	
C9A	1.06731 (16)	0.94255 (17)	0.33170 (15)	0.0321 (4)	
H9A	1.0997	0.9408	0.3909	0.038*	
C10A	1.15646 (17)	0.96339 (19)	0.27366 (15)	0.0331 (5)	
C25A	1.1193 (2)	0.9870 (3)	0.17901 (18)	0.0508 (7)	
H25A	1.0552	1.0224	0.1769	0.076*	
H25B	1.1120	0.9221	0.1472	0.076*	
H25C	1.1675	1.0314	0.1538	0.076*	
C11A	0.99067 (19)	1.0334 (2)	0.3321 (2)	0.0445 (6)	
H11A	1.0268	1.0994	0.3423	0.053*	
H11B	0.9530	1.0377	0.2758	0.053*	
C12A	0.91727 (19)	1.0190 (2)	0.4008 (2)	0.0430 (6)	
H12A	0.8670	1.0747	0.3949	0.052*	
H12B	0.9537	1.0257	0.4574	0.052*	
C13A	0.86364 (16)	0.91191 (19)	0.39464 (15)	0.0333 (5)	
H13A	0.8248	0.9089	0.3380	0.040*	
C14A	0.94152 (16)	0.81979 (18)	0.39779 (15)	0.0330 (4)	
C27A	1.00401 (19)	0.8173 (2)	0.48636 (16)	0.0434 (6)	
H27A	0.9595	0.8234	0.5316	0.065*	
H27B	1.0509	0.8751	0.4900	0.065*	
H27C	1.0404	0.7519	0.4925	0.065*	
C15A	0.88247 (19)	0.7147 (2)	0.38937 (19)	0.0433 (6)	
H15A	0.8452	0.7119	0.3328	0.052*	
H15B	0.9303	0.6567	0.3929	0.052*	
C16A	0.8090 (2)	0.6995 (2)	0.4580 (2)	0.0475 (6)	
H16A	0.8473	0.6887	0.5134	0.057*	
H16B	0.7698	0.6361	0.4447	0.057*	
C17A	0.73689 (17)	0.7918 (2)	0.46578 (16)	0.0391 (5)	
C18A	0.78804 (17)	0.90099 (19)	0.46337 (15)	0.0348 (5)	
H18A	0.8214	0.9178	0.5205	0.042*	
C19A	0.69298 (17)	0.9692 (2)	0.44596 (16)	0.0390 (5)	
H19A	0.7106	1.0387	0.4240	0.047*	
C20A	0.63477 (19)	0.9810 (3)	0.52715 (17)	0.0464 (6)	
C29A	0.5409 (2)	1.0501 (3)	0.5060 (2)	0.0584 (8)	
H29A	0.5615	1.1191	0.4889	0.088*	
H29B	0.5039	1.0558	0.5562	0.088*	
H29C	0.4988	1.0185	0.4596	0.088*	
C30A	0.7016 (3)	1.0367 (3)	0.5986 (2)	0.0630 (9)	
H30A	0.7523	0.9885	0.6228	0.095*	
H30B	0.6608	1.0599	0.6430	0.095*	
H30C	0.7338	1.0965	0.5747	0.095*	
C21A	0.6028 (2)	0.8707 (3)	0.5547 (2)	0.0594 (8)	
H21A	0.5843	0.8743	0.6137	0.071*	
H21B	0.5430	0.8498	0.5183	0.071*	
C22A	0.6834 (2)	0.7858 (3)	0.54953 (19)	0.0522 (7)	
H22A	0.6522	0.7170	0.5533	0.063*	
H22B	0.7332	0.7932	0.5987	0.063*	
C28A	0.65841 (19)	0.8003 (2)	0.38829 (19)	0.0474 (6)	
H28A	0.6862	0.7752	0.3363	0.057*	
H28B	0.5992	0.7585	0.3977	0.057*	
O3A	1.48053 (13)	1.00257 (17)	0.26184 (14)	0.0501 (5)	
H3A1	1.5240	0.9707	0.2923	0.075*	
O19A	0.63221 (13)	0.91049 (17)	0.38001 (12)	0.0462 (4)	
C1B	−0.2473 (3)	0.2089 (3)	0.1513 (3)	0.0712 (11)	
H1B1	−0.2525	0.1906	0.0904	0.085*	
H1B2	−0.2003	0.1599	0.1813	0.085*	
C2B	−0.3505 (3)	0.1957 (3)	0.1856 (3)	0.0715 (10)	
H2B1	−0.3991	0.2409	0.1532	0.086*	
H2B2	−0.3729	0.1232	0.1776	0.086*	
C3B	−0.3458 (2)	0.2240 (3)	0.2808 (2)	0.0570 (8)	
H3B	−0.2984	0.1756	0.3125	0.068*	
C4B	−0.3095 (2)	0.3373 (2)	0.29996 (19)	0.0486 (6)	
C23B	−0.3896 (2)	0.4190 (3)	0.2711 (3)	0.0698 (9)	
H23D	−0.4115	0.4082	0.2111	0.105*	
H23E	−0.3616	0.4885	0.2788	0.105*	
H23F	−0.4462	0.4117	0.3050	0.105*	
C24B	−0.2904 (3)	0.3460 (4)	0.3991 (2)	0.0769 (11)	
H24D	−0.2740	0.4177	0.4147	0.115*	
H24E	−0.2352	0.3008	0.4190	0.115*	
H24F	−0.3501	0.3250	0.4253	0.115*	
C5B	−0.20754 (18)	0.3516 (2)	0.25790 (17)	0.0408 (5)	
H5B	−0.1617	0.3012	0.2890	0.049*	
C6B	−0.1576 (2)	0.4583 (3)	0.2765 (2)	0.0610 (8)	
H6B1	−0.1625	0.4771	0.3365	0.073*	
H6B2	−0.1924	0.5119	0.2406	0.073*	
C7B	−0.0463 (2)	0.4541 (3)	0.2579 (2)	0.0583 (8)	
H7B1	−0.0113	0.4045	0.2976	0.070*	
H7B2	−0.0165	0.5231	0.2693	0.070*	
C8B	−0.02884 (19)	0.4216 (2)	0.16479 (19)	0.0418 (5)	
C26B	−0.0611 (3)	0.5157 (3)	0.1055 (3)	0.0720 (11)	
H26D	−0.0521	0.4977	0.0466	0.108*	
H26E	−0.0203	0.5761	0.1223	0.108*	
H26F	−0.1309	0.5318	0.1108	0.108*	
C9B	−0.09107 (18)	0.3195 (2)	0.14203 (15)	0.0388 (5)	
H9B	−0.0595	0.2651	0.1803	0.047*	
C10B	−0.20469 (19)	0.3209 (2)	0.16231 (17)	0.0444 (6)	
C25B	−0.2713 (2)	0.3952 (4)	0.0998 (2)	0.0739 (11)	
H25D	−0.2462	0.3944	0.0436	0.111*	
H25E	−0.2685	0.4659	0.1222	0.111*	
H25F	−0.3400	0.3708	0.0953	0.111*	
C11B	−0.0740 (2)	0.2824 (3)	0.05069 (18)	0.0598 (9)	
H11C	−0.1128	0.2186	0.0376	0.072*	
H11D	−0.0979	0.3361	0.0093	0.072*	
C12B	0.0367 (2)	0.2607 (3)	0.0419 (2)	0.0569 (8)	
H12C	0.0450	0.2402	−0.0172	0.068*	
H12D	0.0583	0.2018	0.0790	0.068*	
C13B	0.10390 (18)	0.3549 (2)	0.06550 (15)	0.0394 (5)	
H13B	0.0829	0.4111	0.0244	0.047*	
C14B	0.08792 (18)	0.39761 (19)	0.15696 (16)	0.0372 (5)	
C27B	0.1273 (2)	0.3165 (3)	0.22556 (17)	0.0516 (7)	
H27D	0.1914	0.2897	0.2115	0.077*	
H27E	0.0799	0.2593	0.2267	0.077*	
H27F	0.1350	0.3497	0.2813	0.077*	
C15B	0.1532 (2)	0.4977 (2)	0.1723 (2)	0.0571 (8)	
H15C	0.1288	0.5515	0.1312	0.069*	
H15D	0.1453	0.5244	0.2299	0.069*	
C16B	0.2653 (2)	0.4786 (3)	0.1634 (2)	0.0636 (9)	
H16C	0.2925	0.4347	0.2111	0.076*	
H16D	0.3005	0.5457	0.1678	0.076*	
C17B	0.2866 (2)	0.4255 (2)	0.07837 (19)	0.0492 (6)	
C18B	0.21616 (19)	0.3323 (2)	0.05480 (15)	0.0396 (5)	
H18B	0.2384	0.2703	0.0891	0.048*	
C19B	0.2399 (2)	0.3173 (3)	−0.03868 (18)	0.0620 (9)	
H19B	0.1868	0.2753	−0.0703	0.074*	
C20B	0.3440 (3)	0.2659 (4)	−0.0460 (2)	0.0688 (10)	
C29B	0.3596 (4)	0.2538 (6)	−0.1423 (3)	0.115 (2)	
H29D	0.3569	0.3220	−0.1694	0.173*	
H29E	0.3073	0.2097	−0.1695	0.173*	
H29F	0.4242	0.2221	−0.1482	0.173*	
C30B	0.3482 (4)	0.1572 (4)	−0.0041 (4)	0.0938 (14)	
H30D	0.3997	0.1158	−0.0277	0.141*	
H30E	0.2840	0.1228	−0.0155	0.141*	
H30F	0.3635	0.1644	0.0572	0.141*	
C21B	0.4246 (2)	0.3413 (4)	−0.0031 (2)	0.0659 (9)	
H21C	0.4882	0.3036	0.0068	0.079*	
H21D	0.4347	0.3992	−0.0423	0.079*	
C22B	0.3958 (2)	0.3857 (3)	0.0823 (2)	0.0608 (8)	
H22C	0.4411	0.4435	0.0995	0.073*	
H22D	0.4051	0.3313	0.1262	0.073*	
C28B	0.2655 (3)	0.4934 (3)	−0.0014 (3)	0.0750 (11)	
H28C	0.2104	0.5418	0.0062	0.090*	
H28D	0.3250	0.5341	−0.0122	0.090*	
O3B	−0.4427 (2)	0.2113 (2)	0.3131 (2)	0.0794 (8)	
H3B1	−0.4599	0.1493	0.3090	0.119*	
O19B	0.2390 (2)	0.4238 (3)	−0.07185 (16)	0.0825 (9)	

### Atomic displacement parameters (Å^2^)

**Table:**

	*U*^11^	*U*^22^	*U*^33^	*U*^12^	*U*^13^	*U*^23^
C1A	0.0370 (12)	0.0286 (11)	0.0573 (15)	−0.0037 (9)	0.0131 (11)	0.0015 (10)
C2A	0.0405 (13)	0.0349 (12)	0.0575 (15)	−0.0081 (10)	0.0151 (11)	0.0023 (11)
C3A	0.0307 (11)	0.0408 (13)	0.0431 (12)	−0.0083 (10)	0.0061 (9)	0.0012 (10)
C4A	0.0303 (10)	0.0366 (12)	0.0424 (12)	−0.0021 (9)	0.0068 (9)	−0.0029 (10)
C23A	0.0501 (15)	0.0683 (19)	0.0447 (14)	−0.0031 (14)	0.0178 (12)	−0.0087 (13)
C24A	0.0324 (12)	0.0425 (14)	0.079 (2)	0.0015 (11)	0.0106 (12)	0.0039 (13)
C5A	0.0277 (10)	0.0303 (11)	0.0350 (11)	−0.0034 (9)	0.0039 (8)	−0.0024 (9)
C6A	0.0332 (11)	0.0328 (12)	0.0571 (15)	−0.0037 (9)	0.0087 (10)	−0.0124 (11)
C7A	0.0317 (11)	0.0273 (11)	0.0655 (16)	−0.0036 (9)	0.0124 (11)	−0.0051 (11)
C8A	0.0259 (10)	0.0328 (11)	0.0406 (11)	−0.0017 (8)	0.0022 (8)	−0.0032 (9)
C26A	0.0348 (12)	0.0666 (18)	0.0418 (13)	−0.0087 (12)	0.0005 (9)	−0.0081 (12)
C9A	0.0286 (10)	0.0286 (11)	0.0392 (11)	−0.0003 (8)	0.0041 (8)	0.0003 (9)
C10A	0.0316 (10)	0.0313 (11)	0.0368 (11)	0.0005 (9)	0.0043 (8)	0.0042 (9)
C25A	0.0412 (13)	0.0647 (19)	0.0463 (14)	0.0044 (13)	0.0030 (11)	0.0171 (13)
C11A	0.0365 (12)	0.0308 (12)	0.0681 (17)	0.0038 (10)	0.0145 (12)	0.0092 (12)
C12A	0.0349 (12)	0.0298 (12)	0.0662 (17)	−0.0004 (10)	0.0155 (11)	−0.0025 (11)
C13A	0.0276 (10)	0.0326 (11)	0.0399 (12)	−0.0002 (9)	0.0044 (8)	0.0012 (9)
C14A	0.0276 (10)	0.0288 (10)	0.0426 (12)	−0.0007 (8)	0.0038 (8)	0.0003 (9)
C27A	0.0361 (11)	0.0497 (15)	0.0440 (13)	0.0024 (11)	0.0018 (9)	0.0052 (12)
C15A	0.0370 (12)	0.0317 (12)	0.0622 (16)	−0.0041 (10)	0.0096 (11)	−0.0029 (11)
C16A	0.0417 (13)	0.0362 (13)	0.0656 (17)	−0.0055 (11)	0.0098 (12)	0.0070 (12)
C17A	0.0315 (11)	0.0414 (13)	0.0448 (13)	−0.0054 (10)	0.0059 (9)	0.0024 (10)
C18A	0.0293 (10)	0.0360 (12)	0.0392 (12)	−0.0017 (9)	0.0039 (9)	−0.0002 (9)
C19A	0.0319 (11)	0.0438 (13)	0.0414 (12)	0.0010 (10)	0.0043 (9)	0.0001 (11)
C20A	0.0374 (12)	0.0576 (17)	0.0452 (14)	0.0042 (12)	0.0104 (10)	−0.0046 (12)
C29A	0.0428 (14)	0.074 (2)	0.0596 (18)	0.0124 (15)	0.0118 (13)	−0.0085 (16)
C30A	0.0633 (19)	0.079 (2)	0.0469 (16)	0.0099 (17)	0.0034 (14)	−0.0166 (15)
C21A	0.0505 (15)	0.069 (2)	0.0625 (18)	−0.0006 (15)	0.0262 (13)	0.0029 (16)
C22A	0.0479 (14)	0.0561 (17)	0.0547 (16)	−0.0056 (13)	0.0173 (12)	0.0106 (13)
C28A	0.0357 (12)	0.0511 (15)	0.0546 (15)	−0.0044 (11)	−0.0004 (10)	−0.0086 (12)
O3A	0.0318 (8)	0.0523 (11)	0.0673 (12)	−0.0081 (8)	0.0104 (8)	0.0076 (10)
O19A	0.0380 (9)	0.0549 (11)	0.0442 (10)	0.0065 (8)	−0.0049 (7)	−0.0054 (8)
C1B	0.064 (2)	0.067 (2)	0.086 (2)	−0.0353 (18)	0.0331 (18)	−0.0368 (19)
C2B	0.0626 (19)	0.066 (2)	0.089 (3)	−0.0335 (17)	0.0255 (18)	−0.0284 (19)
C3B	0.0506 (16)	0.0530 (17)	0.0701 (19)	−0.0124 (14)	0.0211 (14)	−0.0017 (15)
C4B	0.0413 (13)	0.0477 (15)	0.0575 (15)	−0.0065 (12)	0.0084 (11)	−0.0075 (13)
C23B	0.0454 (16)	0.063 (2)	0.104 (3)	0.0024 (15)	0.0206 (17)	0.000 (2)
C24B	0.0607 (19)	0.112 (3)	0.0605 (19)	−0.023 (2)	0.0180 (15)	−0.017 (2)
C5B	0.0352 (11)	0.0368 (12)	0.0504 (14)	−0.0047 (10)	0.0035 (10)	−0.0068 (11)
C6B	0.0501 (16)	0.0507 (17)	0.085 (2)	−0.0141 (14)	0.0227 (15)	−0.0233 (16)
C7B	0.0470 (15)	0.0514 (16)	0.079 (2)	−0.0181 (13)	0.0180 (14)	−0.0327 (15)
C8B	0.0379 (12)	0.0282 (11)	0.0590 (15)	−0.0054 (9)	0.0026 (10)	0.0031 (11)
C26B	0.0490 (16)	0.0510 (18)	0.117 (3)	0.0050 (14)	0.0129 (18)	0.037 (2)
C9B	0.0401 (12)	0.0371 (12)	0.0388 (12)	−0.0108 (10)	0.0009 (9)	−0.0040 (10)
C10B	0.0393 (12)	0.0494 (15)	0.0443 (13)	−0.0125 (11)	0.0024 (10)	−0.0038 (12)
C25B	0.0444 (16)	0.111 (3)	0.065 (2)	−0.0022 (18)	−0.0034 (14)	0.022 (2)
C11B	0.0533 (16)	0.083 (2)	0.0436 (14)	−0.0297 (16)	0.0066 (12)	−0.0196 (15)
C12B	0.0573 (16)	0.0645 (19)	0.0505 (15)	−0.0237 (15)	0.0146 (13)	−0.0230 (14)
C13B	0.0390 (12)	0.0444 (13)	0.0340 (11)	−0.0114 (10)	−0.0013 (9)	0.0048 (10)
C14B	0.0364 (11)	0.0314 (11)	0.0430 (12)	−0.0055 (9)	−0.0002 (9)	−0.0040 (9)
C27B	0.0461 (14)	0.0678 (19)	0.0398 (13)	0.0066 (14)	−0.0015 (10)	0.0051 (13)
C15B	0.0489 (15)	0.0438 (15)	0.080 (2)	−0.0155 (12)	0.0113 (14)	−0.0201 (15)
C16B	0.0478 (15)	0.062 (2)	0.082 (2)	−0.0183 (15)	0.0073 (14)	−0.0259 (17)
C17B	0.0407 (13)	0.0497 (16)	0.0568 (16)	−0.0121 (12)	0.0028 (11)	−0.0006 (13)
C18B	0.0423 (12)	0.0441 (14)	0.0320 (11)	−0.0060 (11)	0.0003 (9)	0.0004 (10)
C19B	0.0491 (15)	0.099 (3)	0.0386 (14)	−0.0242 (17)	0.0072 (11)	−0.0054 (16)
C20B	0.0576 (18)	0.098 (3)	0.0538 (17)	−0.0161 (19)	0.0226 (14)	−0.0196 (18)
C29B	0.083 (3)	0.201 (7)	0.067 (2)	−0.040 (4)	0.036 (2)	−0.049 (3)
C30B	0.090 (3)	0.079 (3)	0.120 (4)	0.003 (2)	0.045 (3)	−0.023 (3)
C21B	0.0448 (15)	0.095 (3)	0.0585 (18)	−0.0074 (17)	0.0080 (13)	0.0002 (18)
C22B	0.0371 (13)	0.080 (2)	0.0638 (18)	−0.0079 (15)	−0.0007 (12)	−0.0097 (17)
C28B	0.0590 (19)	0.069 (2)	0.098 (3)	−0.0090 (17)	0.0142 (19)	0.035 (2)
O3B	0.0670 (14)	0.0738 (16)	0.1036 (19)	−0.0283 (13)	0.0428 (14)	−0.0159 (15)
O19B	0.0687 (15)	0.127 (3)	0.0513 (13)	−0.0092 (16)	0.0022 (11)	0.0403 (15)

### Geometric parameters (Å, °)

**Table:**

C1A—C2A	1.535 (3)	C1B—C2B	1.522 (4)
C1A—C10A	1.546 (3)	C1B—C10B	1.532 (4)
C1A—H1A1	0.9700	C1B—H1B1	0.9700
C1A—H1A2	0.9700	C1B—H1B2	0.9700
C2A—C3A	1.503 (4)	C2B—C3B	1.518 (5)
C2A—H2A1	0.9700	C2B—H2B1	0.9700
C2A—H2A2	0.9700	C2B—H2B2	0.9700
C3A—O3A	1.438 (3)	C3B—O3B	1.431 (4)
C3A—C4A	1.544 (3)	C3B—C4B	1.535 (4)
C3A—H3A	0.9800	C3B—H3B	0.9800
C4A—C23A	1.534 (4)	C4B—C23B	1.523 (5)
C4A—C24A	1.539 (4)	C4B—C24B	1.542 (5)
C4A—C5A	1.560 (3)	C4B—C5B	1.565 (4)
C23A—H23A	0.9600	C23B—H23D	0.9600
C23A—H23B	0.9600	C23B—H23E	0.9600
C23A—H23C	0.9600	C23B—H23F	0.9600
C24A—H24A	0.9600	C24B—H24D	0.9600
C24A—H24B	0.9600	C24B—H24E	0.9600
C24A—H24C	0.9600	C24B—H24F	0.9600
C5A—C6A	1.525 (3)	C5B—C6B	1.521 (4)
C5A—C10A	1.558 (3)	C5B—C10B	1.538 (4)
C5A—H5A	0.9800	C5B—H5B	0.9800
C6A—C7A	1.523 (3)	C6B—C7B	1.532 (4)
C6A—H6A1	0.9700	C6B—H6B1	0.9700
C6A—H6A2	0.9700	C6B—H6B2	0.9700
C7A—C8A	1.537 (3)	C7B—C8B	1.540 (4)
C7A—H7A1	0.9700	C7B—H7B1	0.9700
C7A—H7A2	0.9700	C7B—H7B2	0.9700
C8A—C26A	1.546 (3)	C8B—C26B	1.543 (4)
C8A—C9A	1.560 (3)	C8B—C9B	1.559 (3)
C8A—C14A	1.597 (3)	C8B—C14B	1.595 (3)
C26A—H26A	0.9600	C26B—H26D	0.9600
C26A—H26B	0.9600	C26B—H26E	0.9600
C26A—H26C	0.9600	C26B—H26F	0.9600
C9A—C11A	1.537 (3)	C9B—C11B	1.529 (4)
C9A—C10A	1.572 (3)	C9B—C10B	1.568 (3)
C9A—H9A	0.9800	C9B—H9B	0.9800
C10A—C25A	1.538 (3)	C10B—C25B	1.569 (5)
C25A—H25A	0.9600	C25B—H25D	0.9600
C25A—H25B	0.9600	C25B—H25E	0.9600
C25A—H25C	0.9600	C25B—H25F	0.9600
C11A—C12A	1.517 (4)	C11B—C12B	1.514 (4)
C11A—H11A	0.9700	C11B—H11C	0.9700
C11A—H11B	0.9700	C11B—H11D	0.9700
C12A—C13A	1.532 (3)	C12B—C13B	1.516 (4)
C12A—H12A	0.9700	C12B—H12C	0.9700
C12A—H12B	0.9700	C12B—H12D	0.9700
C13A—C18A	1.534 (3)	C13B—C18B	1.542 (4)
C13A—C14A	1.558 (3)	C13B—C14B	1.551 (3)
C13A—H13A	0.9800	C13B—H13B	0.9800
C14A—C27A	1.543 (3)	C14B—C27B	1.539 (4)
C14A—C15A	1.545 (3)	C14B—C15B	1.543 (3)
C27A—H27A	0.9600	C27B—H27D	0.9600
C27A—H27B	0.9600	C27B—H27E	0.9600
C27A—H27C	0.9600	C27B—H27F	0.9600
C15A—C16A	1.518 (4)	C15B—C16B	1.527 (4)
C15A—H15A	0.9700	C15B—H15C	0.9700
C15A—H15B	0.9700	C15B—H15D	0.9700
C16A—C17A	1.523 (4)	C16B—C17B	1.529 (4)
C16A—H16A	0.9700	C16B—H16C	0.9700
C16A—H16B	0.9700	C16B—H16D	0.9700
C17A—C28A	1.525 (4)	C17B—C28B	1.514 (5)
C17A—C22A	1.537 (4)	C17B—C18B	1.532 (4)
C17A—C18A	1.544 (3)	C17B—C22B	1.533 (4)
C18A—C19A	1.534 (3)	C18B—C19B	1.523 (4)
C18A—H18A	0.9800	C18B—H18B	0.9800
C19A—O19A	1.452 (3)	C19B—O19B	1.444 (5)
C19A—C20A	1.542 (3)	C19B—C20B	1.541 (5)
C19A—H19A	0.9800	C19B—H19B	0.9800
C20A—C30A	1.531 (4)	C20B—C30B	1.522 (7)
C20A—C21A	1.533 (5)	C20B—C29B	1.535 (5)
C20A—C29A	1.535 (4)	C20B—C21B	1.542 (5)
C29A—H29A	0.9600	C29B—H29D	0.9600
C29A—H29B	0.9600	C29B—H29E	0.9600
C29A—H29C	0.9600	C29B—H29F	0.9600
C30A—H30A	0.9600	C30B—H30D	0.9600
C30A—H30B	0.9600	C30B—H30E	0.9600
C30A—H30C	0.9600	C30B—H30F	0.9600
C21A—C22A	1.524 (5)	C21B—C22B	1.519 (5)
C21A—H21A	0.9700	C21B—H21C	0.9700
C21A—H21B	0.9700	C21B—H21D	0.9700
C22A—H22A	0.9700	C22B—H22C	0.9700
C22A—H22B	0.9700	C22B—H22D	0.9700
C28A—O19A	1.442 (4)	C28B—O19B	1.424 (5)
C28A—H28A	0.9700	C28B—H28C	0.9700
C28A—H28B	0.9700	C28B—H28D	0.9700
O3A—H3A1	0.8200	O3B—H3B1	0.8200
			
C2A—C1A—C10A	113.4 (2)	C2B—C1B—C10B	113.4 (3)
C2A—C1A—H1A1	108.9	C2B—C1B—H1B1	108.9
C10A—C1A—H1A1	108.9	C10B—C1B—H1B1	108.9
C2A—C1A—H1A2	108.9	C2B—C1B—H1B2	108.9
C10A—C1A—H1A2	108.9	C10B—C1B—H1B2	108.9
H1A1—C1A—H1A2	107.7	H1B1—C1B—H1B2	107.7
C3A—C2A—C1A	110.3 (2)	C3B—C2B—C1B	110.6 (3)
C3A—C2A—H2A1	109.6	C3B—C2B—H2B1	109.5
C1A—C2A—H2A1	109.6	C1B—C2B—H2B1	109.5
C3A—C2A—H2A2	109.6	C3B—C2B—H2B2	109.5
C1A—C2A—H2A2	109.6	C1B—C2B—H2B2	109.5
H2A1—C2A—H2A2	108.1	H2B1—C2B—H2B2	108.1
O3A—C3A—C2A	107.9 (2)	O3B—C3B—C2B	110.7 (3)
O3A—C3A—C4A	112.9 (2)	O3B—C3B—C4B	108.3 (3)
C2A—C3A—C4A	113.49 (19)	C2B—C3B—C4B	113.3 (3)
O3A—C3A—H3A	107.4	O3B—C3B—H3B	108.2
C2A—C3A—H3A	107.4	C2B—C3B—H3B	108.2
C4A—C3A—H3A	107.4	C4B—C3B—H3B	108.2
C23A—C4A—C24A	107.7 (2)	C23B—C4B—C3B	112.3 (3)
C23A—C4A—C3A	111.4 (2)	C23B—C4B—C24B	107.2 (3)
C24A—C4A—C3A	107.50 (19)	C3B—C4B—C24B	106.4 (3)
C23A—C4A—C5A	115.2 (2)	C23B—C4B—C5B	114.0 (3)
C24A—C4A—C5A	108.6 (2)	C3B—C4B—C5B	107.2 (2)
C3A—C4A—C5A	106.31 (18)	C24B—C4B—C5B	109.6 (2)
C4A—C23A—H23A	109.5	C4B—C23B—H23D	109.5
C4A—C23A—H23B	109.5	C4B—C23B—H23E	109.5
H23A—C23A—H23B	109.5	H23D—C23B—H23E	109.5
C4A—C23A—H23C	109.5	C4B—C23B—H23F	109.5
H23A—C23A—H23C	109.5	H23D—C23B—H23F	109.5
H23B—C23A—H23C	109.5	H23E—C23B—H23F	109.5
C4A—C24A—H24A	109.5	C4B—C24B—H24D	109.5
C4A—C24A—H24B	109.5	C4B—C24B—H24E	109.5
H24A—C24A—H24B	109.5	H24D—C24B—H24E	109.5
C4A—C24A—H24C	109.5	C4B—C24B—H24F	109.5
H24A—C24A—H24C	109.5	H24D—C24B—H24F	109.5
H24B—C24A—H24C	109.5	H24E—C24B—H24F	109.5
C6A—C5A—C10A	110.85 (17)	C6B—C5B—C10B	111.1 (2)
C6A—C5A—C4A	114.69 (19)	C6B—C5B—C4B	113.8 (2)
C10A—C5A—C4A	117.16 (19)	C10B—C5B—C4B	117.9 (2)
C6A—C5A—H5A	104.1	C6B—C5B—H5B	104.1
C10A—C5A—H5A	104.1	C10B—C5B—H5B	104.1
C4A—C5A—H5A	104.1	C4B—C5B—H5B	104.1
C7A—C6A—C5A	109.95 (19)	C5B—C6B—C7B	110.2 (2)
C7A—C6A—H6A1	109.7	C5B—C6B—H6B1	109.6
C5A—C6A—H6A1	109.7	C7B—C6B—H6B1	109.6
C7A—C6A—H6A2	109.7	C5B—C6B—H6B2	109.6
C5A—C6A—H6A2	109.7	C7B—C6B—H6B2	109.6
H6A1—C6A—H6A2	108.2	H6B1—C6B—H6B2	108.1
C6A—C7A—C8A	114.0 (2)	C6B—C7B—C8B	114.4 (2)
C6A—C7A—H7A1	108.8	C6B—C7B—H7B1	108.7
C8A—C7A—H7A1	108.8	C8B—C7B—H7B1	108.7
C6A—C7A—H7A2	108.8	C6B—C7B—H7B2	108.7
C8A—C7A—H7A2	108.8	C8B—C7B—H7B2	108.7
H7A1—C7A—H7A2	107.7	H7B1—C7B—H7B2	107.6
C7A—C8A—C26A	107.2 (2)	C7B—C8B—C26B	107.2 (3)
C7A—C8A—C9A	108.81 (17)	C7B—C8B—C9B	108.3 (2)
C26A—C8A—C9A	112.0 (2)	C26B—C8B—C9B	113.1 (2)
C7A—C8A—C14A	110.28 (19)	C7B—C8B—C14B	110.5 (2)
C26A—C8A—C14A	110.04 (18)	C26B—C8B—C14B	108.8 (2)
C9A—C8A—C14A	108.49 (17)	C9B—C8B—C14B	108.9 (2)
C8A—C26A—H26A	109.5	C8B—C26B—H26D	109.5
C8A—C26A—H26B	109.5	C8B—C26B—H26E	109.5
H26A—C26A—H26B	109.5	H26D—C26B—H26E	109.5
C8A—C26A—H26C	109.5	C8B—C26B—H26F	109.5
H26A—C26A—H26C	109.5	H26D—C26B—H26F	109.5
H26B—C26A—H26C	109.5	H26E—C26B—H26F	109.5
C11A—C9A—C8A	110.67 (18)	C11B—C9B—C8B	110.3 (2)
C11A—C9A—C10A	114.15 (19)	C11B—C9B—C10B	114.3 (2)
C8A—C9A—C10A	117.04 (19)	C8B—C9B—C10B	116.5 (2)
C11A—C9A—H9A	104.5	C11B—C9B—H9B	104.8
C8A—C9A—H9A	104.5	C8B—C9B—H9B	104.8
C10A—C9A—H9A	104.5	C10B—C9B—H9B	104.8
C25A—C10A—C1A	108.3 (2)	C1B—C10B—C5B	107.6 (2)
C25A—C10A—C5A	114.6 (2)	C1B—C10B—C9B	108.6 (2)
C1A—C10A—C5A	106.66 (17)	C5B—C10B—C9B	107.56 (19)
C25A—C10A—C9A	112.66 (19)	C1B—C10B—C25B	107.7 (3)
C1A—C10A—C9A	107.49 (19)	C5B—C10B—C25B	112.6 (3)
C5A—C10A—C9A	106.78 (18)	C9B—C10B—C25B	112.5 (2)
C10A—C25A—H25A	109.5	C10B—C25B—H25D	109.5
C10A—C25A—H25B	109.5	C10B—C25B—H25E	109.5
H25A—C25A—H25B	109.5	H25D—C25B—H25E	109.5
C10A—C25A—H25C	109.5	C10B—C25B—H25F	109.5
H25A—C25A—H25C	109.5	H25D—C25B—H25F	109.5
H25B—C25A—H25C	109.5	H25E—C25B—H25F	109.5
C12A—C11A—C9A	112.3 (2)	C12B—C11B—C9B	111.2 (2)
C12A—C11A—H11A	109.1	C12B—C11B—H11C	109.4
C9A—C11A—H11A	109.1	C9B—C11B—H11C	109.4
C12A—C11A—H11B	109.1	C12B—C11B—H11D	109.4
C9A—C11A—H11B	109.1	C9B—C11B—H11D	109.4
H11A—C11A—H11B	107.9	H11C—C11B—H11D	108.0
C11A—C12A—C13A	112.7 (2)	C11B—C12B—C13B	113.0 (3)
C11A—C12A—H12A	109.0	C11B—C12B—H12C	109.0
C13A—C12A—H12A	109.0	C13B—C12B—H12C	109.0
C11A—C12A—H12B	109.0	C11B—C12B—H12D	109.0
C13A—C12A—H12B	109.0	C13B—C12B—H12D	109.0
H12A—C12A—H12B	107.8	H12C—C12B—H12D	107.8
C12A—C13A—C18A	111.5 (2)	C12B—C13B—C18B	112.5 (2)
C12A—C13A—C14A	110.98 (17)	C12B—C13B—C14B	111.8 (2)
C18A—C13A—C14A	112.58 (19)	C18B—C13B—C14B	111.98 (18)
C12A—C13A—H13A	107.2	C12B—C13B—H13B	106.7
C18A—C13A—H13A	107.2	C18B—C13B—H13B	106.7
C14A—C13A—H13A	107.2	C14B—C13B—H13B	106.7
C27A—C14A—C15A	106.7 (2)	C27B—C14B—C15B	106.8 (2)
C27A—C14A—C13A	110.32 (19)	C27B—C14B—C13B	109.6 (2)
C15A—C14A—C13A	108.20 (17)	C15B—C14B—C13B	108.0 (2)
C27A—C14A—C8A	111.64 (18)	C27B—C14B—C8B	110.4 (2)
C15A—C14A—C8A	111.18 (19)	C15B—C14B—C8B	111.6 (2)
C13A—C14A—C8A	108.73 (18)	C13B—C14B—C8B	110.35 (19)
C14A—C27A—H27A	109.5	C14B—C27B—H27D	109.5
C14A—C27A—H27B	109.5	C14B—C27B—H27E	109.5
H27A—C27A—H27B	109.5	H27D—C27B—H27E	109.5
C14A—C27A—H27C	109.5	C14B—C27B—H27F	109.5
H27A—C27A—H27C	109.5	H27D—C27B—H27F	109.5
H27B—C27A—H27C	109.5	H27E—C27B—H27F	109.5
C16A—C15A—C14A	113.7 (2)	C16B—C15B—C14B	113.2 (2)
C16A—C15A—H15A	108.8	C16B—C15B—H15C	108.9
C14A—C15A—H15A	108.8	C14B—C15B—H15C	108.9
C16A—C15A—H15B	108.8	C16B—C15B—H15D	108.9
C14A—C15A—H15B	108.8	C14B—C15B—H15D	108.9
H15A—C15A—H15B	107.7	H15C—C15B—H15D	107.7
C15A—C16A—C17A	113.9 (2)	C15B—C16B—C17B	113.8 (2)
C15A—C16A—H16A	108.8	C15B—C16B—H16C	108.8
C17A—C16A—H16A	108.8	C17B—C16B—H16C	108.8
C15A—C16A—H16B	108.8	C15B—C16B—H16D	108.8
C17A—C16A—H16B	108.8	C17B—C16B—H16D	108.8
H16A—C16A—H16B	107.7	H16C—C16B—H16D	107.7
C16A—C17A—C28A	112.5 (2)	C28B—C17B—C16B	114.9 (3)
C16A—C17A—C22A	111.7 (2)	C28B—C17B—C18B	100.2 (2)
C28A—C17A—C22A	109.6 (2)	C16B—C17B—C18B	113.0 (2)
C16A—C17A—C18A	113.90 (18)	C28B—C17B—C22B	109.1 (3)
C28A—C17A—C18A	100.9 (2)	C16B—C17B—C22B	110.9 (2)
C22A—C17A—C18A	107.5 (2)	C18B—C17B—C22B	108.2 (3)
C13A—C18A—C19A	113.9 (2)	C19B—C18B—C17B	98.7 (2)
C13A—C18A—C17A	114.4 (2)	C19B—C18B—C13B	114.1 (2)
C19A—C18A—C17A	98.75 (18)	C17B—C18B—C13B	114.0 (2)
C13A—C18A—H18A	109.8	C19B—C18B—H18B	109.9
C19A—C18A—H18A	109.8	C17B—C18B—H18B	109.9
C17A—C18A—H18A	109.8	C13B—C18B—H18B	109.9
O19A—C19A—C18A	103.5 (2)	O19B—C19B—C18B	103.1 (3)
O19A—C19A—C20A	109.8 (2)	O19B—C19B—C20B	110.3 (3)
C18A—C19A—C20A	112.0 (2)	C18B—C19B—C20B	112.6 (3)
O19A—C19A—H19A	110.5	O19B—C19B—H19B	110.2
C18A—C19A—H19A	110.5	C18B—C19B—H19B	110.2
C20A—C19A—H19A	110.5	C20B—C19B—H19B	110.2
C30A—C20A—C21A	112.1 (3)	C30B—C20B—C29B	108.7 (4)
C30A—C20A—C29A	107.6 (3)	C30B—C20B—C19B	110.5 (3)
C21A—C20A—C29A	109.9 (2)	C29B—C20B—C19B	108.2 (3)
C30A—C20A—C19A	109.6 (2)	C30B—C20B—C21B	112.2 (4)
C21A—C20A—C19A	108.1 (2)	C29B—C20B—C21B	109.6 (3)
C29A—C20A—C19A	109.6 (2)	C19B—C20B—C21B	107.5 (3)
C20A—C29A—H29A	109.5	C20B—C29B—H29D	109.5
C20A—C29A—H29B	109.5	C20B—C29B—H29E	109.5
H29A—C29A—H29B	109.5	H29D—C29B—H29E	109.5
C20A—C29A—H29C	109.5	C20B—C29B—H29F	109.5
H29A—C29A—H29C	109.5	H29D—C29B—H29F	109.5
H29B—C29A—H29C	109.5	H29E—C29B—H29F	109.5
C20A—C30A—H30A	109.5	C20B—C30B—H30D	109.5
C20A—C30A—H30B	109.5	C20B—C30B—H30E	109.5
H30A—C30A—H30B	109.5	H30D—C30B—H30E	109.5
C20A—C30A—H30C	109.5	C20B—C30B—H30F	109.5
H30A—C30A—H30C	109.5	H30D—C30B—H30F	109.5
H30B—C30A—H30C	109.5	H30E—C30B—H30F	109.5
C22A—C21A—C20A	114.5 (2)	C22B—C21B—C20B	112.8 (3)
C22A—C21A—H21A	108.6	C22B—C21B—H21C	109.0
C20A—C21A—H21A	108.6	C20B—C21B—H21C	109.0
C22A—C21A—H21B	108.6	C22B—C21B—H21D	109.0
C20A—C21A—H21B	108.6	C20B—C21B—H21D	109.0
H21A—C21A—H21B	107.6	H21C—C21B—H21D	107.8
C21A—C22A—C17A	112.9 (2)	C21B—C22B—C17B	113.4 (3)
C21A—C22A—H22A	109.0	C21B—C22B—H22C	108.9
C17A—C22A—H22A	109.0	C17B—C22B—H22C	108.9
C21A—C22A—H22B	109.0	C21B—C22B—H22D	108.9
C17A—C22A—H22B	109.0	C17B—C22B—H22D	108.9
H22A—C22A—H22B	107.8	H22C—C22B—H22D	107.7
O19A—C28A—C17A	106.4 (2)	O19B—C28B—C17B	107.0 (3)
O19A—C28A—H28A	110.5	O19B—C28B—H28C	110.3
C17A—C28A—H28A	110.5	C17B—C28B—H28C	110.3
O19A—C28A—H28B	110.5	O19B—C28B—H28D	110.3
C17A—C28A—H28B	110.5	C17B—C28B—H28D	110.3
H28A—C28A—H28B	108.6	H28C—C28B—H28D	108.6
C3A—O3A—H3A1	109.5	C3B—O3B—H3B1	109.5
C28A—O19A—C19A	108.55 (19)	C28B—O19B—C19B	108.1 (2)
			
C10A—C1A—C2A—C3A	−58.3 (3)	C10B—C1B—C2B—C3B	−58.4 (5)
C1A—C2A—C3A—O3A	−174.8 (2)	C1B—C2B—C3B—O3B	−179.8 (3)
C1A—C2A—C3A—C4A	59.4 (3)	C1B—C2B—C3B—C4B	58.4 (4)
O3A—C3A—C4A—C23A	−52.3 (3)	O3B—C3B—C4B—C23B	−49.9 (4)
C2A—C3A—C4A—C23A	70.9 (3)	C2B—C3B—C4B—C23B	73.2 (4)
O3A—C3A—C4A—C24A	65.4 (3)	O3B—C3B—C4B—C24B	67.1 (3)
C2A—C3A—C4A—C24A	−171.4 (2)	C2B—C3B—C4B—C24B	−169.8 (3)
O3A—C3A—C4A—C5A	−178.45 (19)	O3B—C3B—C4B—C5B	−175.8 (3)
C2A—C3A—C4A—C5A	−55.3 (3)	C2B—C3B—C4B—C5B	−52.7 (3)
C23A—C4A—C5A—C6A	62.6 (3)	C23B—C4B—C5B—C6B	59.1 (3)
C24A—C4A—C5A—C6A	−58.1 (3)	C3B—C4B—C5B—C6B	−176.0 (3)
C3A—C4A—C5A—C6A	−173.5 (2)	C24B—C4B—C5B—C6B	−61.0 (4)
C23A—C4A—C5A—C10A	−69.9 (3)	C23B—C4B—C5B—C10B	−73.6 (3)
C24A—C4A—C5A—C10A	169.3 (2)	C3B—C4B—C5B—C10B	51.3 (3)
C3A—C4A—C5A—C10A	53.9 (2)	C24B—C4B—C5B—C10B	166.3 (3)
C10A—C5A—C6A—C7A	−63.0 (3)	C10B—C5B—C6B—C7B	−61.3 (3)
C4A—C5A—C6A—C7A	161.6 (2)	C4B—C5B—C6B—C7B	162.8 (3)
C5A—C6A—C7A—C8A	59.0 (3)	C5B—C6B—C7B—C8B	57.5 (4)
C6A—C7A—C8A—C26A	71.5 (3)	C6B—C7B—C8B—C26B	72.7 (3)
C6A—C7A—C8A—C9A	−49.8 (3)	C6B—C7B—C8B—C9B	−49.6 (3)
C6A—C7A—C8A—C14A	−168.72 (19)	C6B—C7B—C8B—C14B	−168.8 (3)
C7A—C8A—C9A—C11A	−178.7 (2)	C7B—C8B—C9B—C11B	−178.6 (2)
C26A—C8A—C9A—C11A	62.9 (3)	C26B—C8B—C9B—C11B	62.7 (3)
C14A—C8A—C9A—C11A	−58.7 (2)	C14B—C8B—C9B—C11B	−58.4 (3)
C7A—C8A—C9A—C10A	48.2 (3)	C7B—C8B—C9B—C10B	49.0 (3)
C26A—C8A—C9A—C10A	−70.2 (2)	C26B—C8B—C9B—C10B	−69.7 (3)
C14A—C8A—C9A—C10A	168.15 (18)	C14B—C8B—C9B—C10B	169.2 (2)
C2A—C1A—C10A—C25A	−70.7 (3)	C2B—C1B—C10B—C5B	52.9 (4)
C2A—C1A—C10A—C5A	53.1 (3)	C2B—C1B—C10B—C9B	169.0 (3)
C2A—C1A—C10A—C9A	167.3 (2)	C2B—C1B—C10B—C25B	−68.8 (4)
C6A—C5A—C10A—C25A	−67.7 (3)	C6B—C5B—C10B—C1B	174.8 (3)
C4A—C5A—C10A—C25A	66.6 (3)	C4B—C5B—C10B—C1B	−51.3 (3)
C6A—C5A—C10A—C1A	172.5 (2)	C6B—C5B—C10B—C9B	58.0 (3)
C4A—C5A—C10A—C1A	−53.2 (2)	C4B—C5B—C10B—C9B	−168.1 (2)
C6A—C5A—C10A—C9A	57.8 (2)	C6B—C5B—C10B—C25B	−66.6 (3)
C4A—C5A—C10A—C9A	−167.93 (18)	C4B—C5B—C10B—C25B	67.3 (3)
C11A—C9A—C10A—C25A	−57.1 (3)	C11B—C9B—C10B—C1B	59.6 (3)
C8A—C9A—C10A—C25A	74.4 (3)	C8B—C9B—C10B—C1B	−169.8 (3)
C11A—C9A—C10A—C1A	62.0 (3)	C11B—C9B—C10B—C5B	175.8 (3)
C8A—C9A—C10A—C1A	−166.42 (19)	C8B—C9B—C10B—C5B	−53.7 (3)
C11A—C9A—C10A—C5A	176.2 (2)	C11B—C9B—C10B—C25B	−59.6 (4)
C8A—C9A—C10A—C5A	−52.3 (2)	C8B—C9B—C10B—C25B	71.0 (3)
C8A—C9A—C11A—C12A	55.8 (3)	C8B—C9B—C11B—C12B	58.7 (3)
C10A—C9A—C11A—C12A	−169.6 (2)	C10B—C9B—C11B—C12B	−167.8 (3)
C9A—C11A—C12A—C13A	−53.6 (3)	C9B—C11B—C12B—C13B	−56.3 (4)
C11A—C12A—C13A—C18A	−178.5 (2)	C11B—C12B—C13B—C18B	−179.0 (2)
C11A—C12A—C13A—C14A	55.1 (3)	C11B—C12B—C13B—C14B	54.0 (3)
C12A—C13A—C14A—C27A	64.8 (3)	C12B—C13B—C14B—C27B	68.1 (3)
C18A—C13A—C14A—C27A	−60.9 (2)	C18B—C13B—C14B—C27B	−59.1 (3)
C12A—C13A—C14A—C15A	−178.8 (2)	C12B—C13B—C14B—C15B	−175.9 (2)
C18A—C13A—C14A—C15A	55.5 (2)	C18B—C13B—C14B—C15B	56.9 (3)
C12A—C13A—C14A—C8A	−57.9 (2)	C12B—C13B—C14B—C8B	−53.6 (3)
C18A—C13A—C14A—C8A	176.36 (18)	C18B—C13B—C14B—C8B	179.1 (2)
C7A—C8A—C14A—C27A	56.9 (3)	C7B—C8B—C14B—C27B	53.5 (3)
C26A—C8A—C14A—C27A	175.0 (2)	C26B—C8B—C14B—C27B	170.9 (3)
C9A—C8A—C14A—C27A	−62.1 (2)	C9B—C8B—C14B—C27B	−65.4 (3)
C7A—C8A—C14A—C15A	−62.1 (2)	C7B—C8B—C14B—C15B	−65.2 (3)
C26A—C8A—C14A—C15A	55.9 (3)	C26B—C8B—C14B—C15B	52.2 (3)
C9A—C8A—C14A—C15A	178.82 (18)	C9B—C8B—C14B—C15B	175.9 (2)
C7A—C8A—C14A—C13A	178.88 (18)	C7B—C8B—C14B—C13B	174.7 (2)
C26A—C8A—C14A—C13A	−63.1 (2)	C26B—C8B—C14B—C13B	−67.8 (3)
C9A—C8A—C14A—C13A	59.8 (2)	C9B—C8B—C14B—C13B	55.9 (3)
C27A—C14A—C15A—C16A	61.8 (3)	C27B—C14B—C15B—C16B	60.5 (3)
C13A—C14A—C15A—C16A	−57.0 (3)	C13B—C14B—C15B—C16B	−57.2 (3)
C8A—C14A—C15A—C16A	−176.3 (2)	C8B—C14B—C15B—C16B	−178.7 (3)
C14A—C15A—C16A—C17A	52.2 (3)	C14B—C15B—C16B—C17B	52.6 (4)
C15A—C16A—C17A—C28A	70.5 (3)	C15B—C16B—C17B—C28B	68.9 (4)
C15A—C16A—C17A—C22A	−165.6 (2)	C15B—C16B—C17B—C18B	−45.2 (4)
C15A—C16A—C17A—C18A	−43.5 (3)	C15B—C16B—C17B—C22B	−166.8 (3)
C12A—C13A—C18A—C19A	71.8 (3)	C28B—C17B—C18B—C19B	44.3 (3)
C14A—C13A—C18A—C19A	−162.71 (19)	C16B—C17B—C18B—C19B	167.1 (3)
C12A—C13A—C18A—C17A	−175.6 (2)	C22B—C17B—C18B—C19B	−69.8 (3)
C14A—C13A—C18A—C17A	−50.1 (3)	C28B—C17B—C18B—C13B	−77.1 (3)
C16A—C17A—C18A—C13A	43.1 (3)	C16B—C17B—C18B—C13B	45.7 (3)
C28A—C17A—C18A—C13A	−77.8 (2)	C22B—C17B—C18B—C13B	168.8 (2)
C22A—C17A—C18A—C13A	167.4 (2)	C12B—C13B—C18B—C19B	67.9 (3)
C16A—C17A—C18A—C19A	164.4 (2)	C14B—C13B—C18B—C19B	−165.3 (2)
C28A—C17A—C18A—C19A	43.6 (2)	C12B—C13B—C18B—C17B	−179.8 (2)
C22A—C17A—C18A—C19A	−71.2 (2)	C14B—C13B—C18B—C17B	−52.9 (3)
C13A—C18A—C19A—O19A	78.2 (2)	C17B—C18B—C19B—O19B	−44.7 (3)
C17A—C18A—C19A—O19A	−43.5 (2)	C13B—C18B—C19B—O19B	76.6 (3)
C13A—C18A—C19A—C20A	−163.6 (2)	C17B—C18B—C19B—C20B	74.2 (3)
C17A—C18A—C19A—C20A	74.6 (2)	C13B—C18B—C19B—C20B	−164.5 (3)
O19A—C19A—C20A—C30A	176.4 (3)	O19B—C19B—C20B—C30B	175.2 (3)
C18A—C19A—C20A—C30A	62.0 (3)	C18B—C19B—C20B—C30B	60.6 (4)
O19A—C19A—C20A—C21A	54.0 (3)	O19B—C19B—C20B—C29B	−65.9 (4)
C18A—C19A—C20A—C21A	−60.4 (3)	C18B—C19B—C20B—C29B	179.5 (4)
O19A—C19A—C20A—C29A	−65.8 (3)	O19B—C19B—C20B—C21B	52.4 (3)
C18A—C19A—C20A—C29A	179.8 (2)	C18B—C19B—C20B—C21B	−62.2 (4)
C30A—C20A—C21A—C22A	−79.1 (3)	C30B—C20B—C21B—C22B	−77.8 (4)
C29A—C20A—C21A—C22A	161.3 (3)	C29B—C20B—C21B—C22B	161.3 (4)
C19A—C20A—C21A—C22A	41.8 (3)	C19B—C20B—C21B—C22B	43.9 (4)
C20A—C21A—C22A—C17A	−44.1 (4)	C20B—C21B—C22B—C17B	−46.1 (5)
C16A—C17A—C22A—C21A	−174.1 (2)	C28B—C17B—C22B—C21B	−47.1 (4)
C28A—C17A—C22A—C21A	−48.6 (3)	C16B—C17B—C22B—C21B	−174.6 (3)
C18A—C17A—C22A—C21A	60.3 (3)	C18B—C17B—C22B—C21B	61.0 (4)
C16A—C17A—C28A—O19A	−151.2 (2)	C16B—C17B—C28B—O19B	−151.1 (3)
C22A—C17A—C28A—O19A	83.8 (3)	C18B—C17B—C28B—O19B	−29.7 (3)
C18A—C17A—C28A—O19A	−29.4 (3)	C22B—C17B—C28B—O19B	83.7 (3)
C17A—C28A—O19A—C19A	2.1 (3)	C17B—C28B—O19B—C19B	1.7 (4)
C18A—C19A—O19A—C28A	26.6 (3)	C18B—C19B—O19B—C28B	27.5 (3)
C20A—C19A—O19A—C28A	−93.1 (2)	C20B—C19B—O19B—C28B	−93.0 (3)

### Hydrogen-bond geometry (Å, °)

**Table:**

*D*—H···*A*	*D*—H	H···*A*	*D*···*A*	*D*—H···*A*
O3A—H3A1···O19A^i^	0.82	2.04	2.853 (3)	171
O3B—H3B1···O3A^ii^	0.82	2.12	2.920 (3)	164

Symmetry codes: (i) *x*+1, *y*, *z*; (ii) *x*−2, *y*−1, *z*.
